# Label-free quantitative proteomics reveals regulation of interferon-induced protein with tetratricopeptide repeats 3 (IFIT3) and 5'-3'-exoribonuclease 2 (XRN2) during respiratory syncytial virus infection

**DOI:** 10.1186/1743-422X-8-442

**Published:** 2011-09-20

**Authors:** Nicola Ternette, Cynthia Wright, Holger B Kramer, Mikael Altun, Benedikt M Kessler

**Affiliations:** 1 Henry Wellcome Building for Molecular Physiology, Nuffield Department of Medicine, University of Oxford, Roosevelt Drive, Oxford, OX3 7BN, UK; 2 Department of Physiology, Anatomy & Genetics, University of Oxford, Oxford, UK

**Keywords:** Respiratory syncytial virus, label-free quantitative proteomics, mass spectrometry, IFIT3, XRN2

## Abstract

A large quantitative study was carried out to compare the proteome of respiratory syncytial virus (RSV) infected versus uninfected cells in order to determine novel pathways regulated during viral infection. RSV infected and mock-infected HEp2 cells were lysed and proteins separated by preparative isoelectric focussing using offgel fractionation. Following tryptic digestion, purified peptides were characterized using label-free quantitative expression profiling by nano-ultra performance liquid chromatography coupled to electrospray ionisation mass spectrometry with collision energy ramping for all-ion fragmentation (UPLC-MS^E^). A total of 1352 unique cellular proteins were identified and their abundance compared between infected and non-infected cells. Ingenuity pathway analysis revealed regulation of several central cellular metabolic and signalling pathways during infection. Selected proteins that were found regulated in RSV infected cells were screened by quantitative real-time PCR for their regulation on the transcriptional level. Synthesis of interferon-induced protein with tetratricopeptide repeats 3 (IFIT3) and 5'-3'-exoribonuclease 2 (XRN2) mRNAs were found to be highly induced upon RSV infection in a time dependent manner. Accordingly, IFIT3 protein levels accumulated during the time course of infection. In contrast, little variation was observed in XRN2 protein levels, but different forms were present in infected versus non-infected cells. This suggests a role of these proteins in viral infection, and analysis of their function will shed further light on mechanisms of RNA virus replication and the host cell defence machinery.

## Background

Human respiratory syncytial virus (RSV) is a pathogen of the family of Paramyxoviridae, causing severe infection of the lower respiratory tract predominantly in young children and the elderly. It is well recognized to be responsible for the majority of paediatric hospitalizations due to lower respiratory tract illness such as bronchiolitis and pneumonia. Although vaccines have been successfully developed for other members of the Paramyxovirus family such as Measles virus, vaccination against RSV infection remains challenging: RSV induced protective immune responses are short lasting and also show effects of enhancing disease severity of secondary infections [1]. However, specific preventative treatment with monoclonal antibody preparations against the viral fusion (F) surface protein can be given to high risk children during annual epidemic peak periods. Nonetheless, there is evidence that most hospitalized children are completely healthy prior to RSV infection and treatment of high risk patients does not influence numbers of hospitalizations [2]. Hence, the need to further understand mechanisms of virus-host interactions and host immune responses is evident.

RSV is an enveloped virus encasing a single-strand negative RNA genome that encodes a total of 9 structural and 2 non-structural proteins that are present in the infected cells only. The virus infects the upper and lower respiratory epithelium and is transmitted by either virus laden airosols or direct contact with infected mucus secretions. Attachment of the virus particle to the target cell is mediated by the surface glyco (G) protein via binding to glycosaminoglycans on the host cell surface [3-5]. Subsequent fusion of virus and cell membrane is catalyzed by the fusion (F) protein [6]. The nucleocapsid formed by the viral ss(-) RNA genome that is entirely complexed by the nucleocapsid (N) protein, is immediately released to the cell cytoplasm following viral and host cell membrane fusion. Transcription of viral mRNA is initiated immediately after fusion and nucleocapsid release. Whereas transcription and replication of the viral ss(-)RNA genome are catalyzed by the viral RNA dependent RNA polymerase, synthesis of viral proteins is conducted by the host cell translation machinery. The matrix proteins (M, M2-1, M2-2) that form the scaffold of the viral particle, have influence on the viral polymerase activity *in vitro *and have been shown to be the major players in viral assembly and budding processes [7]. The virus also encodes for two non-structural (NS) proteins NS1 and NS2 that are only expressed in the infected cell but are not present in the mature viral particles. Assembly of viral particles occurs presumably at raft locations in the plasma membrane since viral proteins associate with detergent resistant membrane regions [8,9] and lipid raft markers can be detected in viral particles [10,11]. Viral proteins are encoded on the genome in the following manner: 3'-NS1-NS2-N-P-M-SH-G-F-(M2-1/M2-2)-L-5' [12-14] and transcription occurs in a sequential polar fashion from 3' to 5' which leads to a higher abundance of proteins encoded close to the 3'-end in the infected cell.

RSV infection is detected by pattern recognition receptors of the host cell that allow initiation of primary antiviral responses. Viral RNA has been shown to be detected by RIG-I and Toll-like receptor (TLR) 3 [15,16], and RSV-F protein is able to activate TLR4 signalling [17-19] and increase TLR4 expression [20]. Also, RSV has been shown to counterbalance cellular antiviral responses to infection. TLR3 and TLR7 responses have been shown to be disrupted during infection [21] and both NS1 and NS2 have been shown to negatively regulate type I interferon response [22-24]. The NS1 protein can interact with cellular elongin C and cullin 2 to form an E3 ligase complex that directs ubiquitination and degradation of signal transducer and activator of transcription (STAT) 2, a downstream target of the type I interferon signalling cascade [25].

Here we present the results of a label-free quantitative proteomic comparison of the proteome of RSV infected versus mock-infected HEp2 cells. Analysis of this data on a systems level revealed major changes of proteins involved in central cellular signalling and metabolic pathways during infection, in particular biosynthesis and metabolism of proteins. Further validation of two individual proteins that were found regulated was performed using qRT-PCR and immunoblotting techniques.

## Methodology

### Virus infection, purification and titer determination

HEp2 cells were cultured in Dulbecco's Modified Eagle's Medium (DMEM) supplemented with 10% fetal bovine serum (FBS), 2 mM L-glutamine, 10 units/ml Penicillin and 0.1 mg/ml Streptomycin (all components from Sigma-Aldrich) at 37°C and 5% CO_2_.

For RSV (subtype A strain long) stock preparation, cells were infected at a multiplicity of infection (MOI) of 1 in FBS free medium. Cell supernatants were replaced with medium containing 0.5% FBS 4 hours post infection (hpi) to remove remaining inactive viral particles. 48 hpi cells were scraped off the flask and supernatants were cleared by centrifugation for 5 min at 300 g. Virus containing supernatans were immediately sterile filtered (0.45 nm) and subsequently ultracentrifuged at 50,000 g for 2 h at 4°C on a 10% sucrose cushion to pellet viral particles. Virus pellets were resuspended in icecold 10% sucrose in PBS and stored at -80°C [26]. Viral titers were determined in a 96 flat well format by infection of 5000 cells/well using 10-fold serial dilutions of the virus preparations in 100 μl medium containing 0.5% FBS. 48 hpi cells were washed, fixed in methanol and immunostained using a monoclonal antibody directed against RSV-P (clone 3C4) [27] followed by incubation with a secondary anti-mouse Fc antibody coupled to horseradish peroxidase (HRP) produced in goat (Dako). Visualization of bound antibodies was performed using the 3-Amino-9-ethylcarbazole (AEC) staining kit (Sigma). The described purification procedure resulted in viral stock solutions of an average concentration of 5·10^6 ^to 2·10^7 ^plaque forming units (pfu) per ml. Infection of cells for both mass spectrometry analysis and RSV time course experiments was consistently performed at a calculated MOI of 2 using purified viral stocks.

### UPLC-MS^E ^analysis

8·10^6 ^HEp2 cells were either infected with RSV or incubated in infection medium lacking the virus. Cells were lysed 24 hpi in lysis buffer (50 mM Tris, pH 7.4, 150 mM NaCl, 5 mM MgCl_2_, 0.5% Ipegal CA-630, 2 mM DTT, 0.5 mM EDTA) for 15 min at 4°C. Lysates were cleared by centrifugation at 5000 g for 10 min. Total protein content was measured by BCA protein assay (Pierce). A total of 1 mg protein of each sample was separated by one-dimensional isoelectric focussing using an offgel fractionater (Agilent) supplemented with pH 4 to 7 gradient strips. An aliquot of each fraction was analyzed on NuPAGE 4-12% gradient gels (Invitrogen) and proteins were visualized by silver staining (SilverSnap, Pierce). Remaining protein material was precipitated with Methanol/Chloroform [28] and protein pellets obtained were resuspended in 100 μl 6 M Urea in 100 mM Tris, pH 7.8. Following reduction with 10 mM DTT, cystein residues were alkylated using a final concentration of 40 mM iodoacetamide for 30 min at room temperature. Samples were diluted with water to a final urea concentration of 0.67 M and trypsin was added in a 1:20 ratio regarding the total protein content of the sample. Digestion was carried out at 37°C over night. Following acidification with formic acid (0.1% final concentration), obtained tryptic peptides were purified on a C18 reverse phase column (SepPak, Waters), dried and resuspended in 20 μl 2% acetonitrile, 0.1% formic acid in water. Peptides were analyzed using nano-ultra performance liquid chromatography coupled to electrospray ionisation mass spectrometry (UPLC-MS^E^, where E refers to low/high collision energy switching) on a Waters quadrupole time-of-flight (Q-tof) Premier instrument as previously described [29]. Each fraction was run in triplicates to allow determination of the significance of quantitation of detected peptides. Processing of the raw data including deisotoping and deconvolution was performed with Protein Lynx Global Server (PLGS) 2.3 (Waters). For MS^E ^data MS/MS spectra were reconstructed by combining all precursor and fragment masses with identical retention times. The mass accuracy of the raw data was corrected using Glu-fibrinopeptide (200 fmol/μl; 700 nl/min flow rate; 785.8426 Da [M + 2H]^2+^) that was infused into the mass spectrometer as a lock mass during analysis (every 30 seconds). Peptides and regarding proteins were identified by searching the peaklists against a database containing all human UniProt/SwissProt entries [version 2009.04.23; 19713 entries] combined with all NCBI entries for RSV subtype A proteins [2009/05/06; 208 entries] with the following parameters: Minimum fragment ion matches per peptide: 3; minimum fragment ion matches per protein: 7; minimum peptide matches per protein: 1; maximum protein mass: 250000 Da; primary digest reagent: trypsin; missed cleavages: 1; fixed modifications: Carbamidomethyl (C); variable modifications: Oxidation (M); false positive rate: 4%. All protein hits that were identified with a confidence of >95% were included in the quantitative analysis. Identical peptides from each triplicate set per sample were clustered based on mass precision (<10 ppm, typically ca 5 ppm) and a retention time tolerance of <0.25 min using the clustering software included in PLGS 2.3. If two or more distinct proteins shared an identical peptide but were found to be regulated differently, then the quantitation algorithm did not include the peptide in question. In order to allow for this, peptide probabilities are always softened by the PLGS software slightly prior to quantitation. Because of this, the contributions from peptides with even 100% probability of presence were suppressed in order to avoid potential errors in quantitation. Normalisation was performed using the PLGS „auto-normalization" function. The statistical significance of relative expression ratios was calculated using a Monte-Carlo algorithm and expressed as p < 0.05 for down-regulated and 1-p > 0.95 for up-reguated proteins, respectively. Ingenuity pathway analysis (IPA) was performed with a final list of 853 proteins using a regulation cutoff filter of 28%. The significance of cellular functions and pathways relevant in RSV infection were calculated using the right-tailed Fisher Exact Test by considering the number of detected proteins that are regulated in the pathway and the total number of proteins that are associated with that pathway in the Ingenuity Knowledge Base.

### Microscopy

HEp2 cells were infected with RSV or left uninfected and fixed in methanol at different times post infection. Phase contrast images of were taken using a wide-field inverted Nikon TE2000U fluorescence microscope fitted with a 20× long working distance objective and colour video camera.

### SDS PAGE and Immunoblotting

HEp2 cells were infected with RSV or left uninfected and harvested at different time points post infection. Cells were lysed as described above and proteins were separated on NuPAGE 4-12% gradient gels (Invitrogen) and transferred to PVDF membranes for subsequent immunoblotting. Primary antibodies used were: mouse monoclonal αRSV-P (clone 3C4) (diln: 1:250 IF, 1:1000 WB) and mouse monoclonal αRSV-F (clone 18F12) (diln: 1:1000) [27]; goat polyclonal αRSV particles (Abcam, ab20745) (diln: 1:1000); rabbit polyclonal αIFIT3 (Abcam, ab76818) (diln: 1:1000); rabbit polyclonal αXRN2a/b (Abcam, ab72181) (diln: 1:2500); rabbit polyclonal αXRN2a (Abcam, ab72284) (diln: 1:2000). Secondary HRP coupled antibodies were obtained from Dako (diln: 1:10000). HRP coupled αGAPDH (diln: 1:35000) and αβ-actin (diln: 1:25000) were purchased from Sigma. Detected proteins were visualized using enhanced chemiluminescence (ECLplus, Amersham).

### qRT-PCR

HEp2 cells were infected with RSV or left uninfected and harvested at different time points post infection. Total RNA was extracted using the RNeasy Plus Mini Kit (Qiagen). A total of 4.5 μg RNA was reverse trancribed using the ThermoScript™ Reverse Transcriptase (Invitrogen). Quantitative real time PCRs were performed using the QuantiTect SYBR Green RT-PCR Kit (Qiagen). An equivalent of 20 ng cDNA as template was analyzed in triplicates on a ABI StepOnePlus Real-Time PCR System (Applied Biosystems) [30]. Specific primers used derived from http://primerdepot.nci.nih.gov/ were: XRN2a: (5'-TGGATTAGGTTTACTGGCATCA-3'; 5'-GCAAGTACCCGTCCATCATAG-3'); IFIT3: (5'-AAGTTCCAGGTGAAATGGCA-3'; 5'-TCGGAACAGCAGAGACACAG-3'); β-actin: (5'-CCTGGCACCCAGCACAAT-3'; 5'-GGGCCGGACTCGTCATACT-3')

## Results

### Time course analysis of RSV infection reveals completed viral protein synthesis and minor cytopathic effect 24 hpi

**Figure 1:**
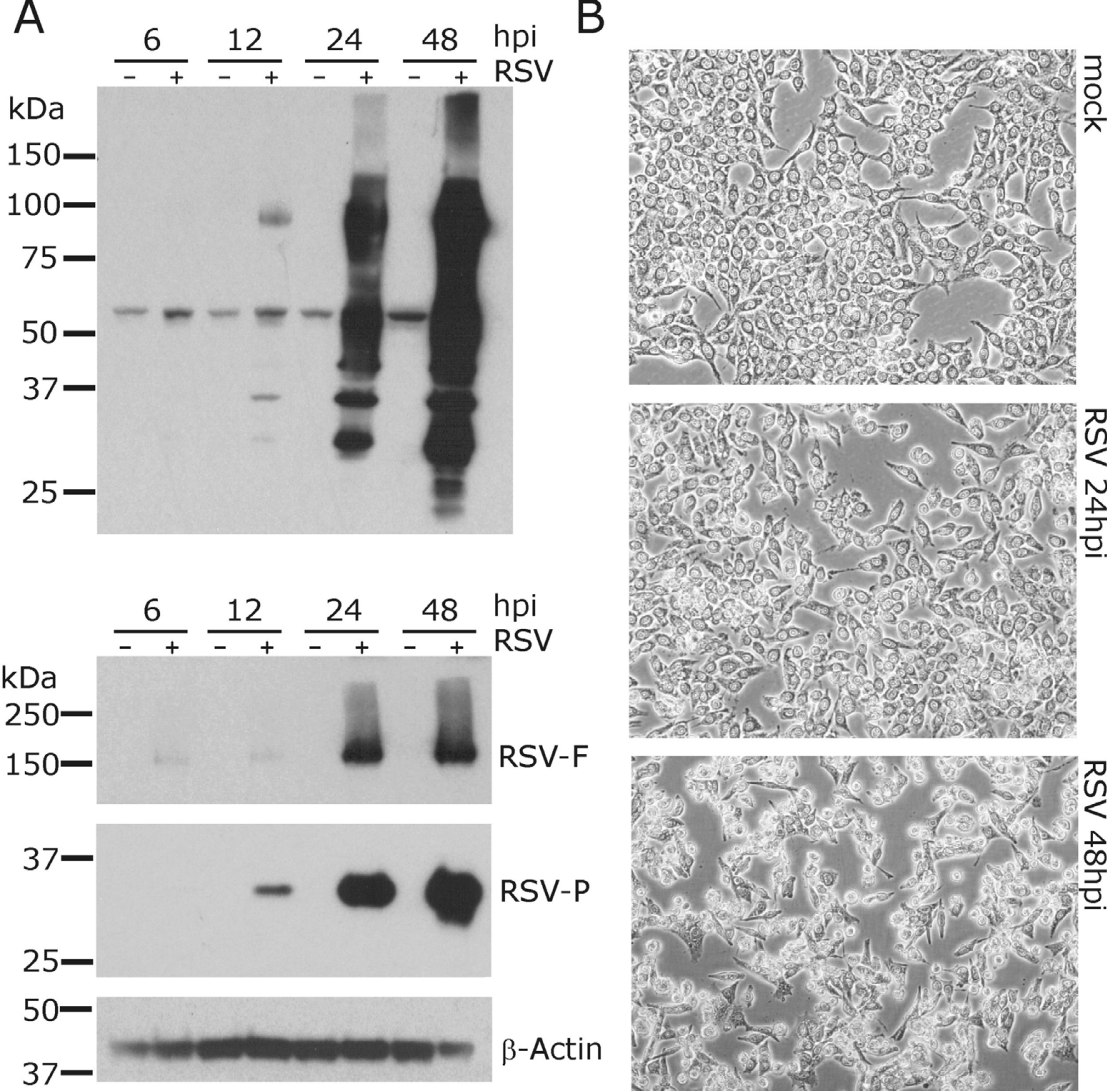
**RSV time course of infection**. HEp2 cells were infected with RSV and harvested at indicated time points post infection. (A) Cells were lysed and proteins analyzed by SDS-PAGE and subsequent immunoblotting using a polyclonal antibody raised against whole RSV particle lysates or monoclonal antibodies directed against the RSV-F and RSV-P protein. (B) Phase contrast images of RSV infected HEp2 cells at indicated time points post infection.

This study aimed to display a final state of the structure of the reprogrammed cell proteome which requires completed synthesis of all viral proteins in the host cell. At the same time, a cytopathic effect (CPE) on the cells caused by viral infection should ideally be minimal. In order to establish the most suitable time point for proteomic analysis, we hence monitored the kinetics of viral protein synthesis and visible CPE during RSV replication by immunoblotting and phase contrast microscopy, respectively. As synthesis of viral components was completed in less than 24 hours post infection (hpi) (Figure 1A) and the visible CPE of infected cells was minor (Figure 1B), this time point was consequently used for the quantitative comparison of the infected and uninfected HEp2 cell proteome.

### Proteomics profiling of RSV infected cells results in identification of 1352 unique cellular and 7 viral proteins

**Figure 2:**
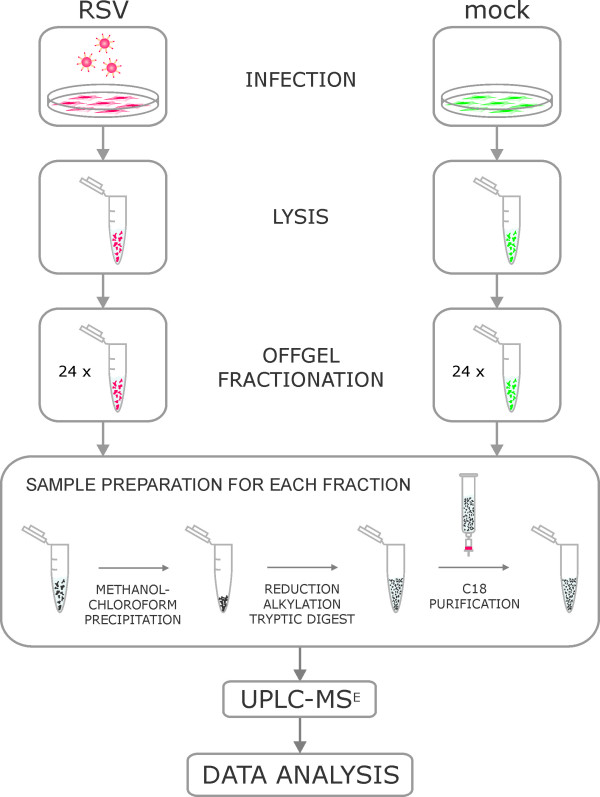
**Label-free profiling workflow**. RSV infected and uninfected HEp2 cells were lysed and proteins were separated by isoelectric focussing using offgel fractionation. Following tryptic digestion, purified peptides were characterized by UPLC-MS^E ^and subsequent relative quantitative expression profiling.

**Figure 3:**
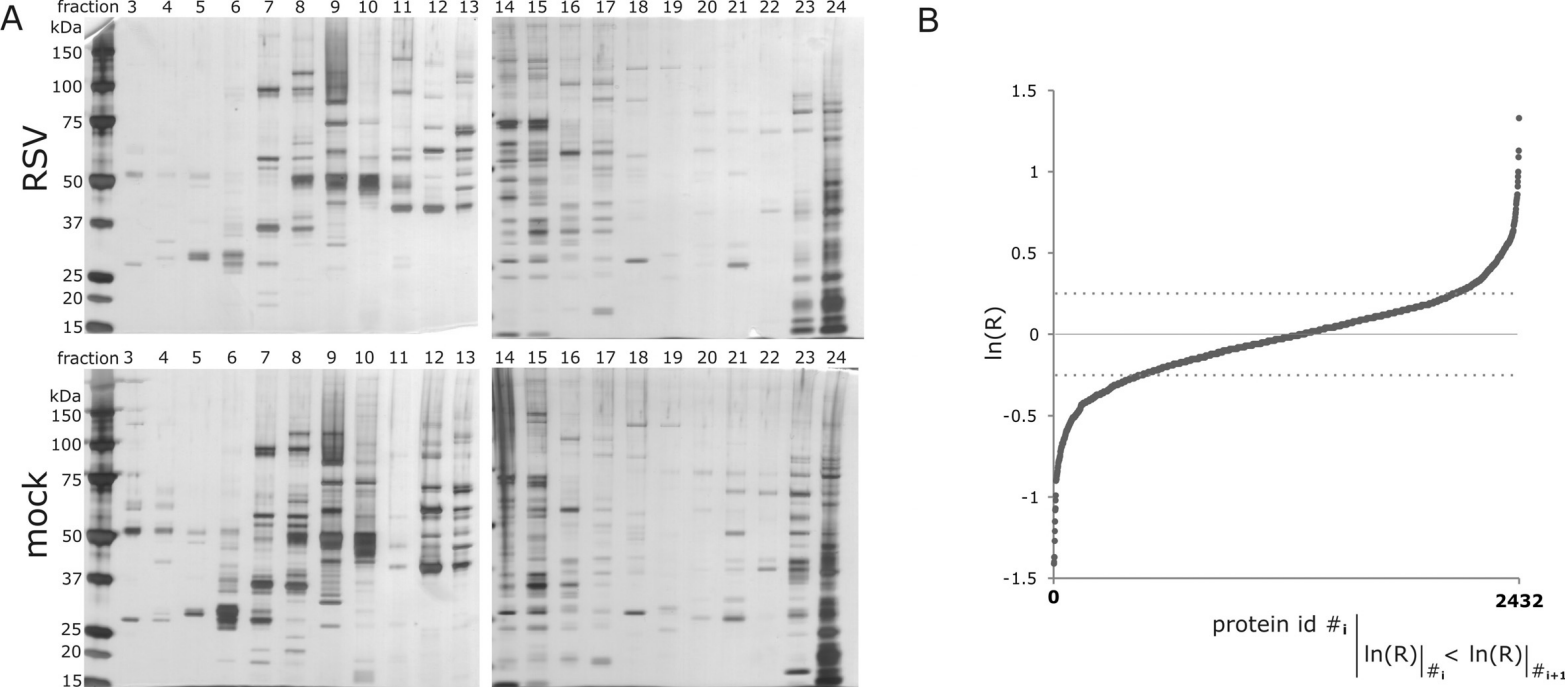
**Quality control of fractionation and obtained quantitative data**. (A) Proteins were separated by SDS-PAGE and visualized by subsequent silver staining. SDS-PAGE analysis of the offgel fractions shows successful separation of protein on the pH strip and indicates differences in the proteome of infected versus uninfected cells. (B) All obtained logarithmic protein expression ratios, ln (R), were plotted in ascending order for each identified protein (protein id) along the x-axis. Dotted horizontal bars indicate the chosen cutoff for up- and down-regulation during RSV infection used in IPA analysis, respectively.

The workflow is summarized in Figure 2. HEp2 cells were either infected with RSV or incubated in infection medium without addition of purified active virus particles. Cells were lysed 24 hpi and proteins were separated by one-dimensional isoelectric focussing using an offgel fractionater into 24 individual fractions each. Each fraction comprises a particular confined pH range and proteins migrate under applied voltage along a pH gradient. A comparable separation of the cellular proteome under both conditions was achieved and confirmed by analysis of an aliquot of the resulting fractions by SDS-PAGE and subsequent silver staining (Figure 3A). All fractions were individually subjected to in-solution trypsin digestion and peptides were purified and analyzed by UPLC-MS^E^. Each fraction was run in triplicates and only protein hits that were confirmed in 2 out of 3 MS runs were included in the analysis [29]. Relative quantitative comparison was performed in a label-free fashion and based on peptide precursor ion intensities [31]. Comparison was carried out between detected proteins of a certain fraction to the regarding control fraction of the same pH range.

We observed identification of many proteins in more than one offgel fraction, which can be explained by two main conditions. Firstly, many proteins occur in multiple isoforms or variants within the cell due to either alternative splicing events of the regarding mRNA, post-transcriptional RNA editing, proteolytic processing and/or post-translational modifications. These variants migrate into distinct fractions during pH-dependent fractionation due to their altered isoelectric point (pI). Secondly, highly abundant proteins might not quantitatively separate into a single fraction but rather distribute throughout neighbouring fractions.

In total, 3134 cellular protein hits of 1352 unique cellular proteins were detected in 20 analyzed fractions. For 2432 protein hits a quantitative expression ratio was calculated (Figure 3B). We identified 7 out of 11 viral proteins that were, as expected, unique to infected cells (table 1).

**Table 1:** RSV proteins identified

NCBI Accession #	Gene name	PLGS score	fr#	r	pep#	% cov
gi|133667|sp|P12579|PHOSP_HRSVL	Phosphoprotein; Short = P	1465.7	4	d	14	47.7
gi|138896|sp|P04544|NS1_HRSVA	Non-structural protein 1; Short = NS1	285.2	14	d	4	61.9
gi|127889|sp|P03418|NCAP_HRSVA	Nucleoprotein; Short = Protein N	346.2	17	e	12	40.4
gi|1353203|sp|P20895|GLYC_HRSVL	Major surface glycoprotein G; Short: Protein G	134.2	17	d	4	27.9
gi|138252|sp|P12568|FUS_HRSVL	Fusion glycoprotein F0; Short = Protein F	324.4	17	d	5	13.8
gi|137260|sp|P04545|M21_HRSVA	Matrix M2-1	584.4	19	e	6	23.3
gi|138727|sp|P03419|MATRX_HRSVA	Matrix protein	266.1	23	f	11	49.2

fr#: Offgel fraction in which the protein was identified with highest score

r: representative replicate of the fraction in which the protein was detected (a,b,c non infected; d,e,f infected)

pep#: number of peptides detected

% cov.: detected peptide coverage of protein sequence in %

### IPA analysis reveals cellular pathways that are interrupted by RSV infection

Ingenuity pathway analysis (IPA) was used for analysis of this data on a systems level. IPA requires expression ratios for all proteins to be included in the analysis. Therefore, proteins that were identified in one condition only had to be assigned a virtual regulation factor. For our dataset the quantitative expression ratio for proteins identified in either condition was set to 3-fold up- or down-regulation [ln(R) = +/- 1.10, were R is the protein expression ratio which is calculated by the sum of peptide intensities in RSV versus mock-infected cells] according to the actual maximal detected experimental regulation values (Figure 3B). In addition, multiple regulation factors obtained for one unique protein detected in distinct fractions had to be reduced to a single value. Since we manually set ratios for proteins that were found in either condition only to ln(R) = +/- 1.10, calculation of an average or mean regulation value could lead to a false emphasis on either condition. Therefore, we decided to delete all multiplicate protein entries that had controversial regulation values throughout different fractions for IPA analysis. As general rule, we deducted multiplicate protein hits if one or more detected logarithmic ratios was of opposite algebraic sign or equal to zero. For remaining multiplicates that showed a common trend for all detected data points, the minimal regulation factor detected was chosen for IPA analysis. Amendment of the data resulted in a final list of 853 proteins, of which 380 were regulated more than 28% (ln(R) = +/- 0.25).

**Figure 4:**
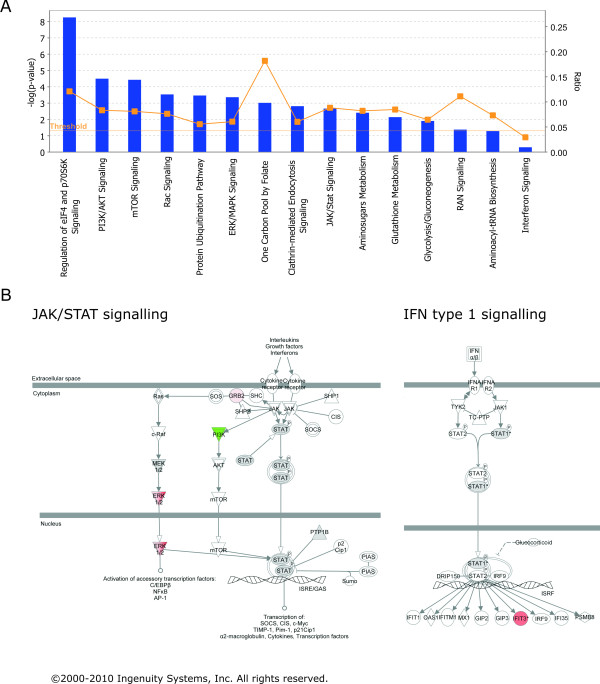
**Canonical pathways regulated during RSV infection**. (A) Selected canonical pathways identified by IPA as regulated during RSV infection plotted by their significance. Yellow data points display the ratio of detected regulated proteins and proteins defined in the pathway. (B) Schematic plots of JAK/STAT and Interferon type I signalling pathways. Proteins that were found regulated during RSV infection are highlighted in green (down-regulated during infection) or red (up-regulated during infection); grey colour indicates regulation below 28%.

Analysis of this amended dataset by IPA associated regulated proteins to cellular functional classes defined by the Ingenuity knowledge database. Disease specific functions included 'respiratory disease' (22 proteins, P-values 5.77·10^-4 ^to 2.06·10^-2^) and 'infectious disease' (31 proteins, P-values 8.64·10^-6 ^to 4.82·10^-2^), in which 25 molecules were correlated to 'infection of cell lines'. 'Protein synthesis' (25 proteins, P-values 8.80·10^-8 ^to 3.31·10^-2^) was noted as most significant cell function affected which reflects the impact of viral infection on exploiting this host cell function to implement protein synthesis. Biosynthesis and metabolism of proteins were the two most influenced subsections of protein synthesis that were regulated. Accordingly, eukaryotic initiation factor 4E (eIF4E) signalling was the cellular pathway found with highest regulation significance. Furthermore, regulation of other central cellular canonical signalling pathways including 'PI3K/Akt signalling', 'mTOR signalling', 'protein ubiquitination pathway', 'ERK/MAPK signalling', and 'RAN signalling' was listed by IPA analysis (Figure 4A). Additionally, multiple metabolic canonical pathways were identified to be impaired by viral infection, including 'one carbon pool by folate', 'aminosugars metabolism', and 'glutathione metabolism' (Figure 4A). Interferon induced 'JAK/STAT signalling' was identified to be differentially regulated by extracellular signal-regulated kinase (ERK) 1 and 2 up-regulation on the one hand and phosphoinositide-3-kinase (PI3K) down-regulation on the other hand (Figure 4B). The central regulatory cellular molecules of selected pathways that were assigned by IPA are listed in table 2. These results reflect the broad influence of RSV infection on major signalling pathways in the cell.

**Table 2:** Overview of regulated proteins and associated pathways in RSV infection

Pathway/ Protein	Swiss Prot #	Gene Name	ln (R)	R	PLGS Score	fr#	r	pep #	% cov
**JAK/STAT**									
GRB2^3^	P62993	growth factor receptor-bound protein 2	0.25	1.28	432.2	17	a	13	71.0
ERK1¹	P27361	extracellular signal-regulated kinase 1	RSV	RSV	326.1	21	e	9	33.8
ERK2¹	P28482	etracellular signal-regulated kinase 2	0.36	1.43	390.6	24	a	18	54.4
PIK3R2^2^	O00459	phosphoinositide-3-kinase, regulatory subunit 2 (beta)	mock	mock	758.6	19	a	23	45.2
**Regulation of eIF4 and p70S6K (includes protein # 1,2,3)**									
EIF1AX	P47813	eukaryotic translation initiation factor 1A, X-linked	-0.43	0.65	346.6	10	a	3	27.8
EIF1AY	O14602	eukaryotic translation initiation factor 1A, Y-linked	-0.37	0.69	367.2	10	a	7	47.2
EIF2B2	P49770	eukaryotic translation initiation factor 2B, subunit 2 beta	-0.35	0.70	476.2	19	a	12	45.9
EIF3C^7^	Q99613	eukaryotic translation initiation factor 3, subunit C	RSV	RSV	268.6	15	e	5	6.4
EIF3E^7^	P60228	eukaryotic translation initiation factor 3, subunit E	-0.40	0.67	462.1	15	a	12	40.2
EIF4E^4^	P06730	eukaryotic translation initiation factor 4E	-0.48	0.62	176.0	17	b	5	31.3
ITGA5^5^	P08648	integrin, alpha 5	0.37	1.45	678.2	4	a	9	10.9
PP2R2B^6^	Q00005	protein phosphatase 2, regulatory subunit B, beta	mock	mock	441.0	19	a	7	22.3
RPS6^7^	P62753	ribosomal protein S6	RSV	RSV	176.7	17	d	5	23.3
**ERK/MAPK (includes protein # 1,2,3,4,5,6)**									
CRKL	P46109	Crk-like protein	mock	mock	308.7	19	a	9	45.5
PAK1^9^	Q13153	p21 protein (Cdc42/Rac)-activated kinase 1	-0.31	0.73	641.9	17	a	9	26.6
PPP1R14B	Q96C90	protein phosphatase 1, regulatory (inhibitor) subunit 14B	-0.59	0.55	270.7	7	a	4	38.1
RAC1^8^	P63000	ras-related C3 botulinum toxin substrate 1	-0.32	0.73	163.5	21	b	3	24.0
RAC2	P15153	ras-related C3 botulinum toxin substrate 2	mock	mock	149.9	21	a	4	31.8
**mTOR (includes protein # 1,2,4,6,7,8)**									
AKT1S1	Q96B36	AKT1 substrate 1 (proline-rich)	-0.29	0.75	163.9	4	a	4	20.7
PLD3	Q8IV08	phospholipase D family, member 3	mock	mock	222.5	22	a	9	19.6
RHOJ	Q9H4E5	ras homolog gene family, member J	-0.38	0.68	150.9	21	b	3	22.9
**AKT (includes protein # 1,2,3,4,5,6)**									
CTNNB1	P35222	catenin (cadherin-associated protein), beta 1	RSV	RSV	507.3	17	e	12	22.3
HLA-B^10^	P30484	major histocompatibility complex, class I, B	-0.38	0.68	465.8	20	b	5	16.6
**RAC (includes protein # 1,2,5,8,9)**									
ARPC5^12^	O15511	actin related protein 2/3 complex, subunit 5	0.27	1.31	333.6	14	a	6	41.7
ARPC5L^12^	Q9BPX5	actin related protein 2/3 complex, subunit 5-like	0.31	1.36	261.0	19	a	7	66.0
IQGAP1	P46940	IQ motif containing GTPase activating protein 1	mock	mock	1092.7	15	a	41	39.2
**RAN**									
CSE1L	P55060	chromosome segregation 1-like protein (exportin 2)	mock	mock	559.9	20	a	17	29.6
IPO5	O00410	importin 5	RSV	RSV	168.6	7	e	8	9.2
**Clathrin mediated Endocytosis (includes protein # 2,3,5,8,12)**									
CTTN	Q14247	cortactin	mock	mock	356.7	12	a	24	42.9
PPP3CA	Q08209	protein phosphatase 3, catalytic subunit, alpha isozyme	-0.30	0.74	357.0	17	a	8	19.8
SH3GLB1	Q9Y371	SH3-domain GRB2-like endophilin B1	mock	mock	349.2	15	a	3	9.9
TSG101	Q99816	tumor susceptibility gene 101	mock	mock	271.2	19	a	6	17.4
**Protein Ubiquitination (includes protein # 10)**									
B2M	P61769	beta-2-microglobulin	0.56	1.75	352.1	21	a	3	37.8
BAG1	Q99933	BCL2-associated athanogene	mock	mock	515.4	10	a	14	47.8
DNAJA1	P31689	DnaJ (Hsp40) homolog, subfamily A, member 1	RSV	RSV	233.5	24	d	13	36.8
DNAJB11	Q9UBS4	DnaJ (Hsp40) homolog, subfamily B, member 11	0.25	1.28	601.3	19	a	12	43.0
HLA-C	Q95604	major histocompatibility complex, class I, C	-0.35	0.70	390.6	20	b	7	30.9
PSMC5	P62195	proteasome 26S subunit, ATPase, 5	RSV	RSV	355.9	24	d	15	42.6
PSMD5	Q16401	proteasome 26S subunit, non-ATPase, 5	-0.32	0.73	1246.8	12	a	26	41.4
SKP1	P63208	S-phase kinase-associated protein 1	-0.32	0.73	999.1	4	a	9	46.6
TCEB1	Q15369	transcription elongation factor B (SIII), pp 1 (elongin C)	-0.29	0.75	630.9	5	a	7	66.1
TCEB2	Q15370	transcription elongation factor B (SIII), pp 2 (elongin B)	RSV	RSV	163.7	5	d	8	89.8
UBE2F	Q969M7	ubiquitin-conjugating enzyme E2F	RSV	RSV	118.4	22	e	4	22.2
UBE2G2	P60604	ubiquitin-conjugating enzyme E2G 2	mock	mock	120.4	5	b	3	27.9
USP11	P51784	ubiquitin specific peptidase 11	-0.32	0.73	643.5	10	a	30	49.2
**Interferon**									
IFIT3	O14879	Interferon-induced protein with tetratricopeptide repeats 3	1.09	2.97	441.8	10	b	14	48.2
**Glycolysis**									
ACSS2^11^	Q9NR19	acyl-CoA synthetase short-chain family member 2	mock	mock	277.5	21	a	8	17.1
ALDH1A1	P00352	aldehyde dehydrogenase 1 family, member A1	RSV	RSV	370.0	17	d	7	30.5
ALDH1A2	O94788	aldehyde dehydrogenase 1 family, member A2	RSV	RSV	281.6	17	d	9	31.5
ALDH1A3	P47895	aldehyde dehydrogenase 1 family, member A3	RSV	RSV	396.4	23	d	15	49.6
GALK1^13^	P51570	galactokinase 1	-0.31	0.73	1066.4	19	a	17	55.6
PKLR	P30613	pyruvate kinase, liver and RBC	RSV	RSV	515.0	17	d	7	16.6
**One Carbon Pool by Folate**									
GCSH	P23434	glycine cleavage system protein H	mock	mock	189.5	4	a	3	25.4
MTHFD1	P11586	methylenetetrahydrofolate dehydrogenase (NADP+ dep.) 1	mock	mock	695.6	24	a	27	39.3
MTR	Q99707	5-methyltetrahydrofolate-homocysteine methyltransferase	mock	mock	1141.5	14	b	35	36.5
TYMS	P04818	thymidylate synthetase	-0.26	0.77	338.3	24	a	12	38.3
**Aminosugars Metabolism (includes protein # 13)**									
GFPT2	O94808	glutamine-fructose-6-phosphate transaminase 2	RSV	RSV	654.1	23	d	16	33.4
HEXA	P06865	hexosaminidase A (alpha polypeptide)	-0.37	0.69	160.6	7	a	8	14.4
HEXB	P07686	hexosaminidase B (beta polypeptide)	mock	mock	265.4	22	a	10	16.7
NAGK	Q9UJ70	N-acetylglucosamine kinase	-0.55	0.58	324.8	17	a	12	52.9
PDE12	Q6L8Q7	phosphodiesterase 12	RSV	RSV	310.9	17	d	13	26.3
**Aminoacyl tRNA Biosynthesis**									
FARSA	Q9Y285	phenylalanyl-tRNA synthetase, alpha subunit	RSV	RSV	243.5	24	d	12	29.5
KARS	Q15046	lysyl-tRNA synthetase	-0.28	0.76	651.6	7	a	24	38.4
SARS2	Q9NP81	seryl-tRNA synthetase 2, mitochondrial	0.57	1.77	626.4	23	a	22	51.0
**Gluthathion Metabolism (includes protein # 11)**									
GCLM	P48507	glutamate-cysteine ligase, modifier subunit	-0.54	0.58	398.5	16	a	7	32.8
GSTM2	P28161	glutathione S-transferase mu 2	RSV	RSV	222.9	12	e	10	43.6
GSTM3	P21266	glutathione S-transferase mu 3	-0.25	0.78	372.1	12	a	13	61.3
GSTO1	P78417	glutathione S-transferase omega 1	-0.4	0.67	645.9	19	a	16	67.2
**OTHER**									
XRN2	Q9H0D6	5'-3' exoribonuclease 2	RSV	RSV	532.2	17	e	17	24.7

1: ERK1, ERK2; 2: PI3KR2; 3: GRB2; 4: EIF4E; 5: ITGA5; 6:PPP2R2B; 7: EIF3C, EIF3E; 8: RAC1; 9: PAK1; 10: HLA-B; 11: ACSS2; 12: ARPC5, ARPC5L; 13:GALK1

R: Protein expression ratio. Calculated by the mean of corresponding peptide intensities detected in infected versus mock-infected cells. 'RSV' or 'mock' stated in this column indicates that the protein was detected in infected cells (RSV) or mock-infected cells (mock) only.

fr#: Offgel fraction in which the protein was identified

r: representative replicate of the fraction in which the protein was detected

pep#: number of peptides detected

% cov.: detected peptide coverage of protein sequence in %

### IFIT3 and XRN2 mRNA synthesis is up-regulated during RSV infection in a time dependent manner

Two individual proteins that were up-regulated in infected cells were further analyzed for dynamics of regulation during infection. Firstly, a target gene of Interferon type I signalling, Interferon stimuated protein with tetratricopeptide repeats 3 (IFIT3) (490 aa; 56,0 kDa), was found up-regulated 3-fold in infected cells (Figure 4B, table 2). IFIT3 is induced upon interferon stimulation and has been described regulated during several RNA viral infections in microarray analyses [32-36]. A second interesting target which was detected in infected cells only is 3'-5' exoribonuclease 2 (XRN2) (table 2). XRN2 has been described to participate in cellular transcription termination [37-39] and processing of ribosomal RNAs [40]. There are two known isoforms of XRN2, namely XRN2a (950 aa; 108,6 kDa), which is the fully translated protein, and XRN2b (874 aa; 100,0 kDa), that lacks the first N-terminal 76 amino acids.

**Figure 5:**
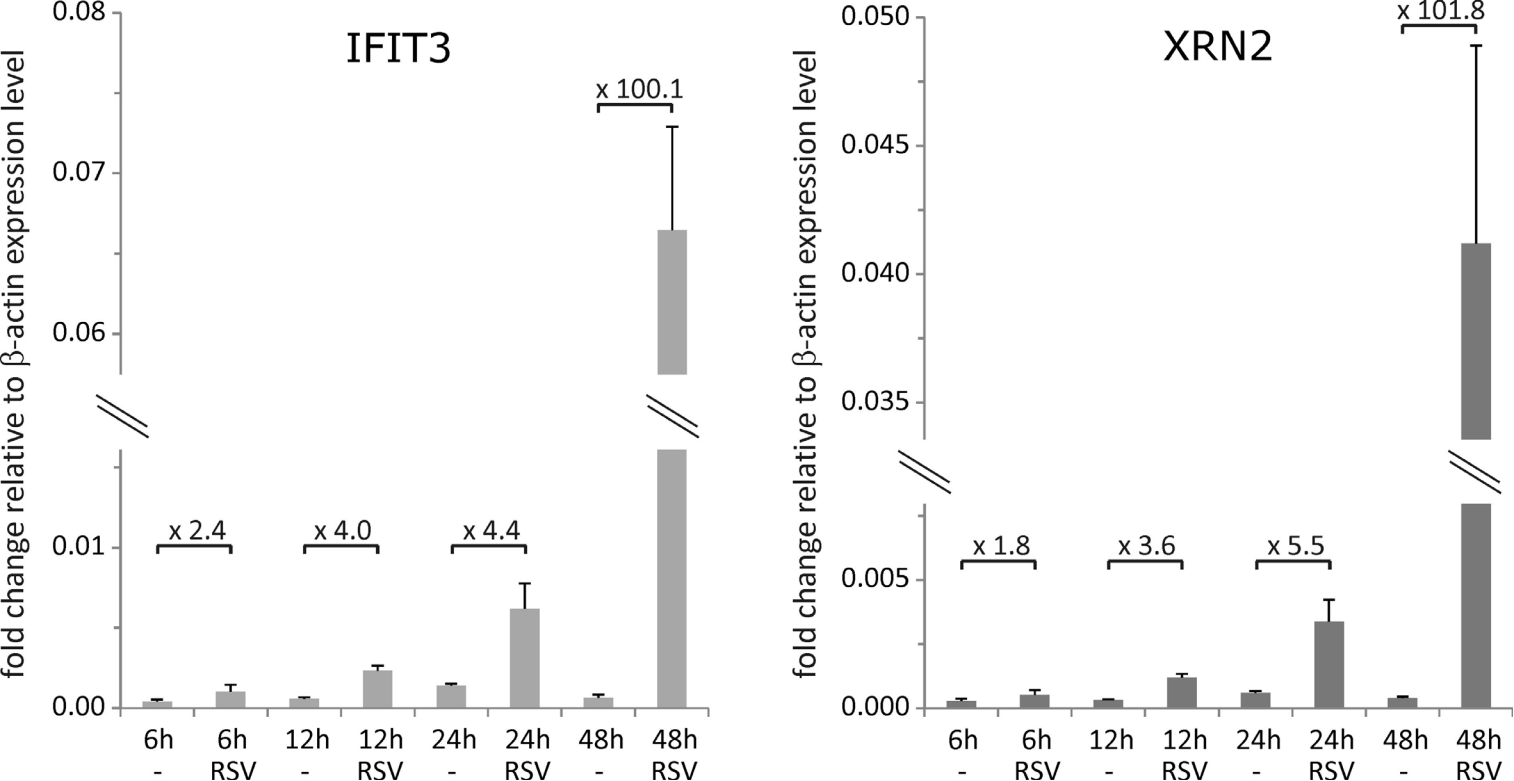
**Transcription of XRN2 and IFIT3 mRNA is induced during RSV infection**. HEp2 cells were infected with RSV or left uninfected (-) for indicated durations. Total RNA extracts were reverse transcribed and analyzed by qRT-PCR. Specific RNA expression levels were measured for IFIT3 and XRN2 and normalized to β-actin mRNA expression levels. Numbers above horizontal bars indicate n-fold change of the RNA expression level detected in infected cells relative to mock-infected cells.

To determine the mRNA expression levels of these two proteins during RSV infection, we performed quantitative real-time PCR (qRT-PCR) experiments of RNA extracted from cells that were harvested at several different time points post RSV infection. IFIT3 and XRN2a mRNAs were found to accumulate up to approximately 100-fold during RSV infection in a time dependent manner (Figure 5).

### Immunoblotting confirmes up-regulation of IFIT3 and suggests the presence of modified XRN2 during RSV infection

**Figure 6:**
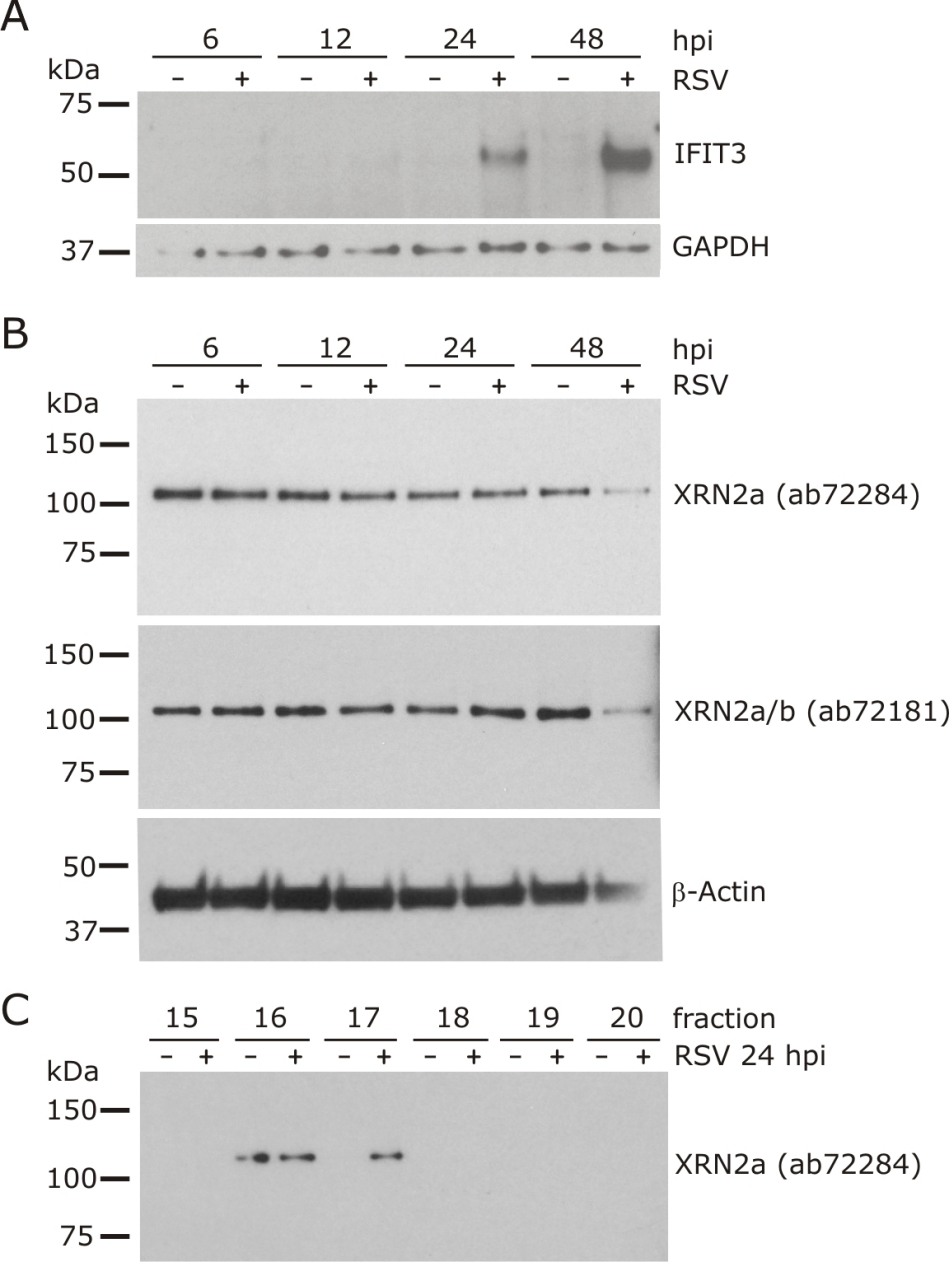
**XRN2 and IFIT3 expression during RSV infection**. HEp2 cells were infected with RSV (+) or left uninfected (-) and harvested at indicated time points post infection. Cells were lysed and proteins analyzed by SDS-PAGE and subsequent immunoblotting using polyclonal antibody raised against IFIT3 (A) or either XRN2a or XRN2a/b (B). Western blot analysis of indicated offgel fractions of a whole cell lysate of RSV infected cells 24 hpi using a polyclonal antibody raised against XRN2a (C).

According to the accumulation of IFIT3 mRNA, analysis of whole cell lysates by immunoblotting also confirmed accumulation of IFIT3 protein during infection over time (Figure 6A). To our surprise, immunoblotting using antibodies directed against either the C- (ab72181) or N-terminus (ab72284) of XRN2 led to detection of a single band of approximately 110 Da that was expressed in constant amounts throughout the time course of infection (Figure 6B). According to the molecular weight detected, we concluded that the detected isoform is XRN2a. We further analyzed the distribution of XRN2 throughout the individual offgel fractions and confirmed the presence of XRN2 in fraction 17 exclusively in infected cells in agreement with the MS results. In addition, equal levels of XRN2 in both mock-infected and infected cells were detected in the neighbouring fraction 16 (Figure 6C). We therefore conclude that XRN2 is most likely modified during RSV infection in a way that results in a pI shift of the protein to a slightly more basic pH. At the same time, the electrophoretic mobility of XRN2 is not notably altered by this modification and thereby escapes detection in unfractionated whole cell lysate analyses.

## Discussion

Recently, proteomic approaches have been exploited to investigate changes in the cellular proteome during RSV infection. A study that analyzed nuclear extracts of RSV infected cells by two-dimensional gel electrophoresis and subsequent MALDI-TOF MS analysis identified regulation and reorganisation of proteins associated with the nuclear domain 10 (ND10), in particular up-regulation of TAR DNA binding protein and reorganisation of promyelocytic leukemia protein (PML) and speckled 100 kDa protein (Sp100) [41]. Additionally, heat shock protein 70 (Hsp70) redistribution and accumulation to the nucleus was described upon RSV infection. Interestingly, Hsp70 has been shown to also relocate into lipid raft membrane structures during RSV infection [42] and has been found to be incorporated into viral particles [43]. In our study, we concentrated on cytoplasmic fractions of infected cells and therefore did not detect the described changes in ND10 composition. However, we identified Hsp70 in 12 distinct offgel fractions of which 11 fractions showed higher abundance of the protein in infected cells. This data supports the hypothesis that RSV infection leads to redistribution of Hsp70 into the cytoplasm (data not shown). A SILAC study of cytoplasmic and nuclear cell extracts of RSV infected lung carcinoma cells (A549) validated changes in ND10 structures and furthermore highlighted novel changes in mitochondrial protein expression patterns [44]. Changes in the disease associated cellular functions 'respiratory disease' and 'infectious disease' and the cellular pathway 'RAN signalling' were observed according to our results. An independent study using 2-D Fluorescence Difference Gel Electrophoresis (2-D DIGE) and subsequent MS analysis revealed similar cellular proteome changes upon infection with different Paramyxoviruses. This study emphasizes the influence of viral infection on induction of apoptosis and the cellular stress response [45].

We used a label-free quantitative proteomic approach to analyze changes of the cellular proteome during RSV infection. In order to improve identification of low abundance proteins, we separated complex whole cell lysates by protein offgel fractionation prior to MS analysis. Our findings emphasize the benefits and challenges of quantitative data analysis using this approach. Since to date quantitation is limited to each fraction separately, obtained relative protein expression ratios reflect the change in abundance of a subpopulation of a protein group that inherits a confined isoelectric point. This approach can therefore generally improve the dataset of studies that aim to include regulation of protein modification in addition to changes in total protein abundance. This could be advantageous for analysis of the influence of RNA virus infection on the cellular proteome, since novel viral proteins are introduced to the cellular environment and immediate changes are likely to occur on the posttranslational level. In particular, detection of the modulation of XRN2 protein during RSV infection would have escaped detection in other quantitative proteome studies since total XRN2 protein levels were constant throughout infection. However, the extent to which this complex dataset can be interpreted by global expression analysis tools like IPA is limited and requires manual sorting of the data prior to analysis, as otherwise median or average values are calculated for multiple obtained expression values of a single protein and the actual regulation of that protein would be obliterated or even falsified. In this study, we excluded most protein hits for which multiplicate expression ratios were obtained from IPA analysis to ensure integrity of the imported dataset. This analysis resulted in identification of several central cellular pathways that were impaired by RSV infection.

It is known that RSV infection leads to activation of ERK1/2 [46], which is dependent on activation of TLR4 signalling and p38 mitogen-activated protein kinase early in respiratory virus infection [47]. Additionally, it was demonstrated that activation of ERK1/2 is required for viral entry using Sendai virus-like particles as a model for Paramyxovirus infection [48]. In this study, ERK1 was identified in RSV infected cells only, and ERK2 was detected with higher abundance in infected cells. Since published results suggest that the levels of the inactive isoforms of ERK1/2 stay constant throughout viral replication [48], we hypothesize that our data may reflect the levels of the active phosphorylated isoforms of both kinases.

The Ubiquitination Pathway was also shown to be highly impaired during RSV replication. It was shown that RSV NS1 protein can recruit elongin C and cullin 2 to direct STAT2 ubiquitination and degradation [25]. Reduced levels of elongin C observed in our dataset might represent a cellular response to counterbalance STAT2 ubiquitination and degradation. Moreover, we detected ubiquitin specific protease 11 (USP11) with lower abundance in infected cells. Interestingly, antiviral activity of USP11 was recently described in influenza A virus replication. Monoubiquitination of the viral nucleoprotein (NP) was shown to be required for efficient replication. USP11 was able to bind and deubiquitinate NP, thereby antagonizing viral replication [49]. Furthermore, the deubiquitinating enzyme OTUB1 was detected with higher abundance in infected cells, indicating a role of this protein in viral infection.

IFIT3 was classified as a member of the interferon (IFN) inducible protein family based on its structural homology to other members and the common clustering of these genes at chromosome 10q23.3 [50,51]. Expression of the IFIT3 gene is regulated by two IFN-stimulated response elements (ISRE) upstream of the TATA box in its promoter region and is induced upon IFNα stimulation [51-53]. IFIT3 has been identified as key mediator in IFNα mediated antiproliferative responses by enhancing both p21 and p27, two negative regulators of cell cycle progression that control transition from G1 to the S phase. A novel mechanism of IFIT3 activation in a STAT1 independent manner by either a STAT2/IRF9 complex lacking STAT1 or by IRF1 alone was proposed recently [54]. However, since STAT2 levels have been shown to be significantly lowered during RSV infection [25], the mechanism of the observed induction of IFIT3 transcription during RSV infection remains to be determined. Interestingly, our data also shows lower levels of STAT1 in infected cells, as it was detected down-regulated during infection in two fractions (fr. 16: mock-infected cells only, fr 17: ln(R) = -0.12). In Figure 4B, STAT1 is indicated as not regulated, since amendment of the data let to inclusion of the minimal regulation factor, and the identification in fraction 16 of mock-infected cells only was discarded for IPA analysis. Antiviral activity of IFIT3 was demonstrated recently, as the growth of several RNA viruses was enhanced in cells in which IFIT3 expression was reduced by siRNA transfection [55,56]. Furthermore, IFIT1, a member of the IFIT family, can recognize 5'-triphosphate RNA and has been shown to form a complex with both IFIT2 and IFIT3 [56]. Therefore it will be of great interest to study the functionality of IFIT3 in further detail.

While the immediate function of IFIT3 remains unknown, XRN2 exerts an exoribonuclease activity that is involved in the torpedo model of polymerase II transcriptional termination [37-39]. Recently, evidence for its role in yeast polymerase I transcription termination has been provided [57]. Additionally, human XRN2 has been shown to be essential for ribosomal RNA maturation and degradation [40]. Linkeage of the XRN2 protein to immune pathways has been given by it's interaction with the Toll interacting rotein TOLLIP [58] that plays an inhibitoy role in Toll-like receptor signalling.

We observed accumulation of XRN2 mRNA levels during the time course of RSV infection in a time dependent manner. In contrast, subsequent immunoblotting experiments in cell extracts using antibodies against the two known isoforms of XRN2 did not confirm an increase in protein levels during infection. However, our data indicates that XRN2 undergoes specific modification(s) during RSV infection that lead to a shift of the protein towards a more basic pI. Possible explanations for the discrepancy between mRNA and protein levels are repression of mRNA processing, enhanced protein turnover or a confined cellular localization of XRN2. Clearly, this warrants further investigation as our data provides evidence for tight regulation and of XRN2 during the time course of RSV infection.

## Conclusion

Quantitative analysis of the proteome of RSV infected versus uninfected cells by UPLC-MSE resulted in identification of 1352 unique cellular proteins. IPA analysis revealed several cellular pathways that are interrupted by viral infection. Further analysis of IFIT3 and XRN2 that were found to be up-regulated during infection were validated on the transcriptional level. While IFIT3 protein levels accumulated accordingly, XRN2 protein expression was constant but showed modification(s) unique to infected cells. In summary, analysis of the specific functions of both IFIT3 and XRN2 during RNA viral replication will be of great value to further unravel mechanisms of RNA virus replication and the cellular antiviral response.

## Competing interests

The authors declare that they have no competing interests.

## Authors' contributions

N.T. performed RSV propagation, purification and infection experiments, RT-PCR and immunoblotting. Sample preparation and proteomics data analysis were performed by N.T. and C.W.. H.B.K. was responsible for the LC-MS/MS instrument setup and sample runs. M.A. supported RT-PCR experiment design and data analysis. B.M.K., C.W. and N.T. developed the study design. B.M.K. supervised this study and revised the manuscript written by N.T.. All authors read and approved the final manuscript.
